# Supplementary material for: Interruption of lactate uptake by inhibiting mitochondrial pyruvate transport unravels direct antitumor and radiosensitizing effects
Source: Nat Commun. 2018 Mar 23;9:1208. doi: 10.1038/s41467-018-03525-0 (PMC5865202; doi:10.1038/s41467-018-03525-0)
Supplement: Supplementary file 1 — Supplementary Information(PDF 2346 kb) [file 41467_2018_3525_MOESM1_ESM.pdf]

Supplementary Information

Interruption of lactate uptake by inhibiting mitochondrial pyruvate transport unravels direct antitumor and radiosensitizing effects

Corbet et al.

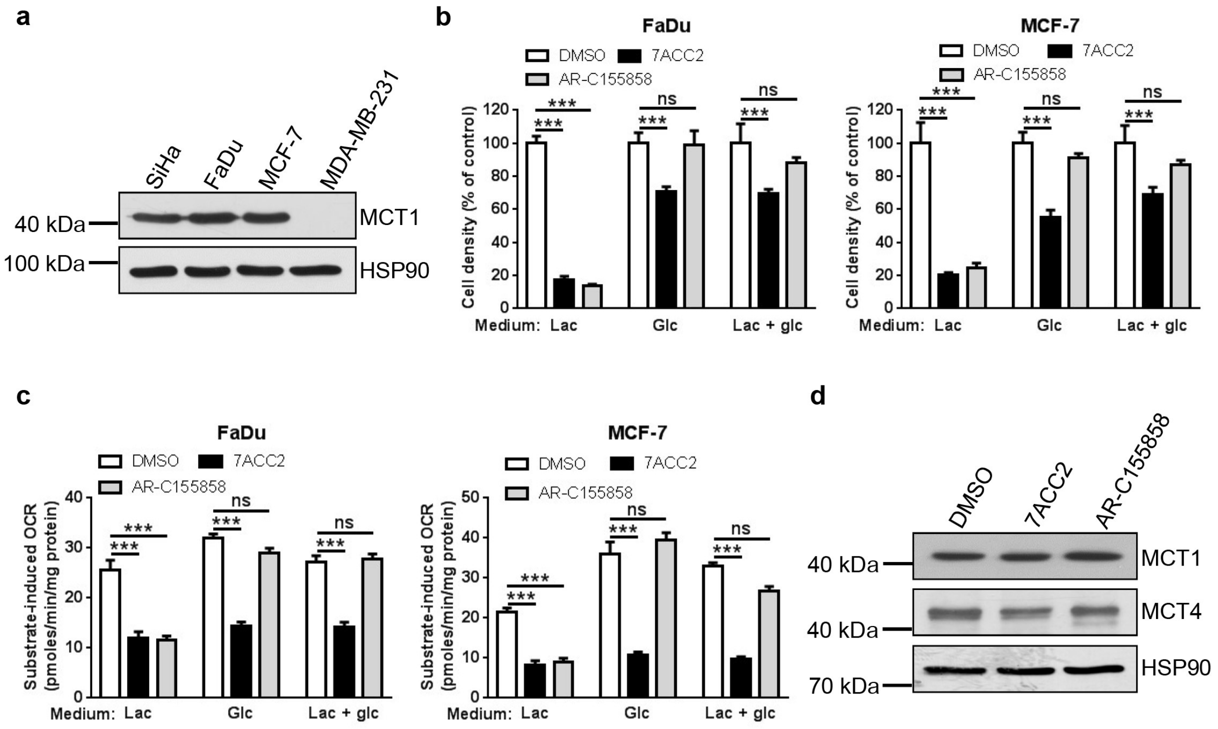

Supplementary Figure 1: 7ACC2 and AR-C155858 exert differential effects on cancer cell growth and respiration depending on glucose availability. (**a**) Representative immunoblotting for MCT1 in the indicated human cancer cell lines; MDA-MB-231 breast cancer cell line was used as a negative control. (**b**) Cell growth (72h) and (**c**) oxygen consumption rate (OCR) of FaDu and MCF-7 cells after treatment with 10 µM 7ACC2 or AR-C155858 in indicated media. (**d**) Representative immunoblotting for MCT1 and MCT4 in SiHa cells after treatment with 10 µM 7ACC2 or AR-C155858 for 24h. Data are represented as mean ± SEM of three independent experiments (with ≥ 6 technical replicates). Significance was determined by two-way ANOVA with Bonferroni multiple-comparison analysis. ***$p<0.001$; ns, not significant.

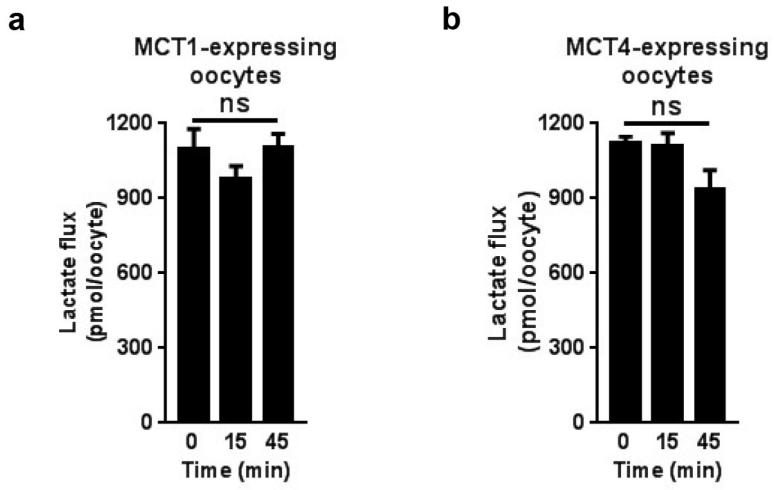

Supplementary Figure 2: 7ACC2 inhibits lactate influx in a MCT-independent manner. ^{14}C-lactate uptake in *Xenopus* oocytes expressing (**a**) MCT1 or (**b**) MCT4 after treatment with 10 µM 7ACC2 for the indicated time periods (n=10 for each group). Data are represented as mean ± SEM. Significance was determined by one-way ANOVA with Bonferroni multiple-comparison analysis. ns, not significant.

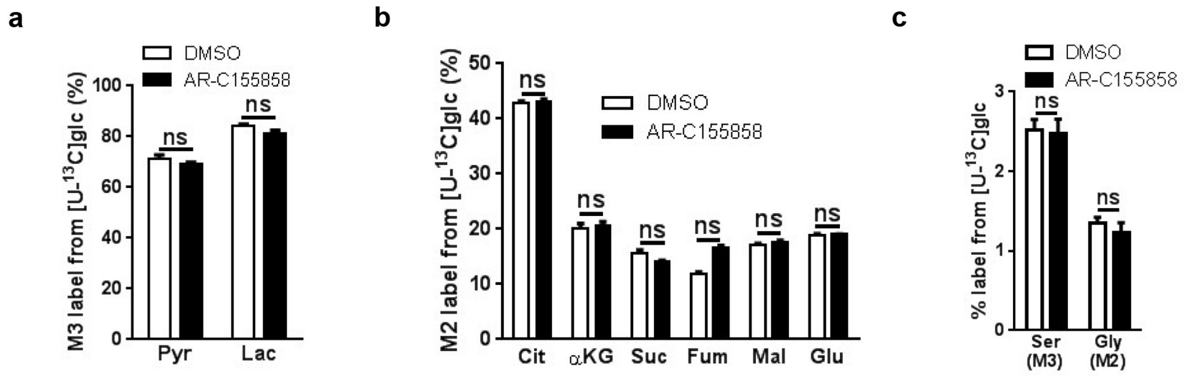

Supplementary Figure 3: MCT1 inhibitor AR-C155858 does not significantly affect glucose metabolism. (**a**) Relative abundance of glycolysis-derived pyruvate and lactate metabolites, (**b**) TCA cycle intermediates and (**c**) serine synthesis pathway metabolites in SiHa cells treated or not with 1 μM AR-C155858 for 24h. Data are represented as mean ± SEM (n=3). Significance was determined by two-way ANOVA with Bonferroni multiple-comparison analysis. ns, not significant.

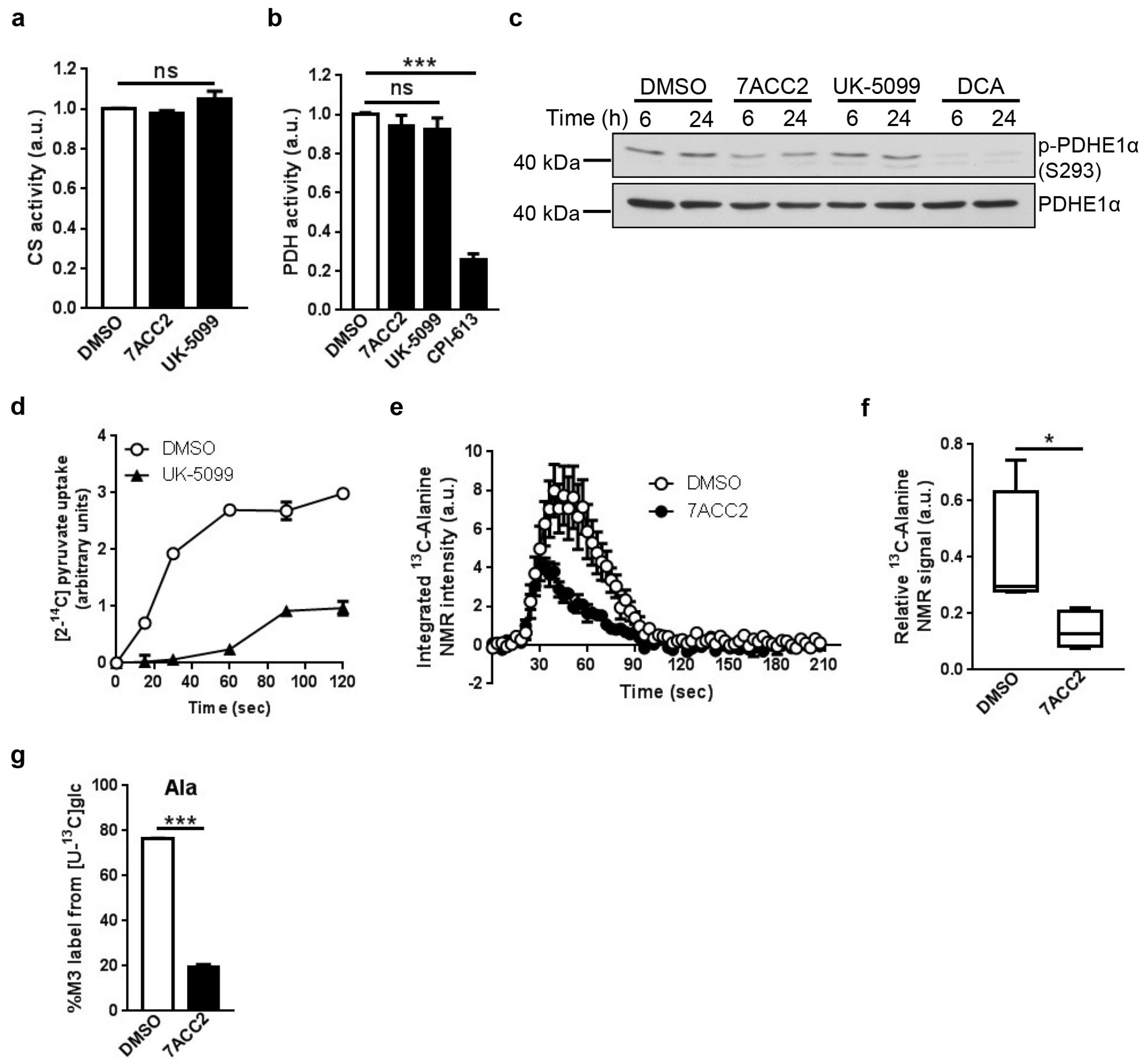

Supplementary Figure 4: 7ACC2 inhibits mitochondrial pyruvate transport, while not altering citrate synthase or pyruvate dehydrogenase activities. Measurements of (**a**) citrate synthase and (**b**) pyruvate dehydrogenase activities in SiHa cells treated with 10 µM 7ACC2, 10 µM UK-5099 or 100 µM CPI-613 for 24h. (**c**) Representative immunoblotting for total and phosphorylated forms of PDH in SiHa cells treated with 10 µM 7ACC2, 10 µM UK-5099 or 5 mM DCA; DCA is used as a positive control. (**d**) [2-^{14}C]pyruvate uptake in isolated mitochondria from SiHa cells treated with 10 µM UK-5099 at different times. (**e**) Time course of ^{13}C-alanine detection and (**f**) relative ^{13}C-alanine NMR signals in control and 7ACC2-treated SiHa tumor xenografts (n=3 for each group). (**g**) Relative abundance of [U-^{13}C]glucose-derived alanine in SiHa cells after treatment with 10 µM 7ACC2 for 24h. Data are represented as mean ± SEM of three independent experiments (with ≥ 6 technical replicates). Significance was determined by student's *t* test (**f, g**) or one-way ANOVA (**a, b**) with Bonferroni multiple-comparison analysis. *p<0.05; ***p<0.001; ns, not significant.

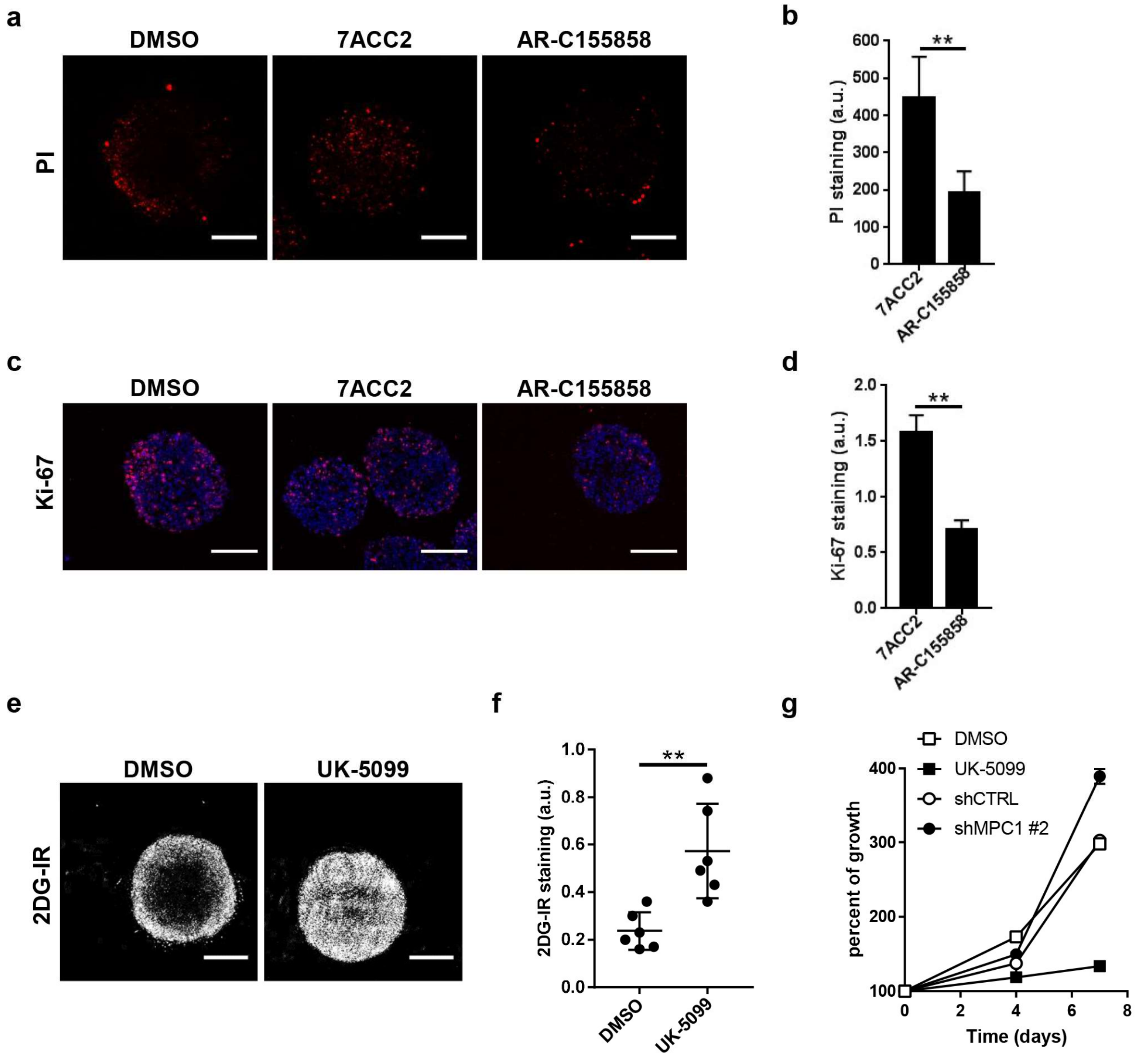

Supplementary Figure 5: 7ACC2 and ARC155858 reduce the growth of tumor spheroids through distinct mechanisms. Representative immunofluorescent pictures and corresponding quantification of (**a-b**) propidium iodide and (**c-d**) Ki-67 stainings in FaDu spheroids treated with 20 µM 7ACC2 or AR-C155858. Scale bars: 200 µm. Data are represented as mean ± SEM of three independent experiments (with ≥ 6 technical replicates). (**e**) Representative immunofluorescence pictures of 2-deoxyglucose-IRDye (2DG-IR, 3h exposure) and **(f)** accumulation within the core (as determined in Fig. 5g) of FaDu spheroids pre-exposed to 20 µM UK-5099 for 24h. Scale bars: 200 µm. (**g**) Time-dependent growth of FaDu spheroids upon treatment with 20 µM UK-5099 for 7 days or initiated from MPC1-targeting shRNA (*vs.* control shRNA). Significance was determined by student's *t* test. **p<0.01.

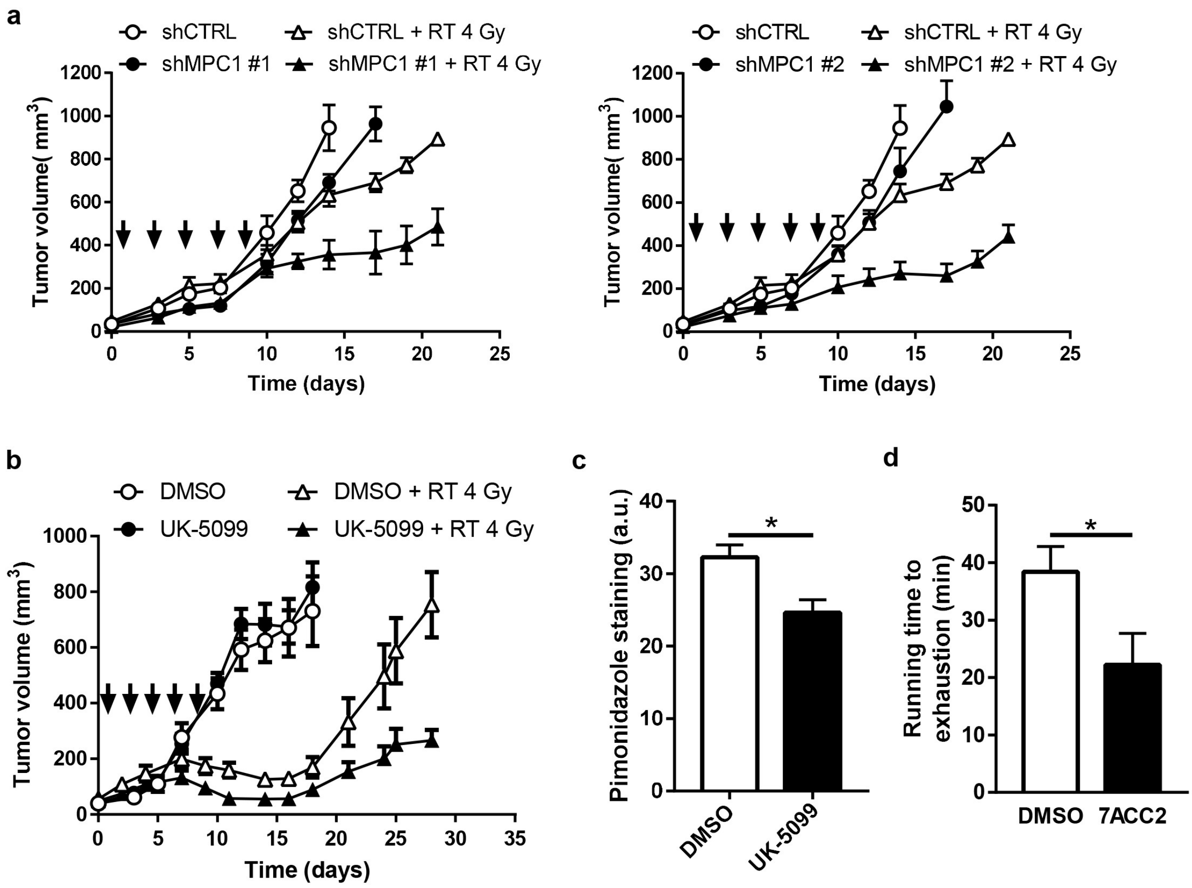

Supplementary Figure 6: MPC1 blockade by genetic knockdown or UK-5099 treatment radiosensitizes tumors. (a) Tumor growth of FaDu xenografts in nude mice resulting from cancer cells expressing two distinct MPC1-targeting shRNA (*vs.*control shRNA) treated with 5 fractions of 4 Gy; arrows indicate the days of irradiation (n=8 for each group). (**b**) Tumor growth of SiHa xenografts in nude mice treated for 2 hours with 3 mg/kg UK-5099 before a 4 Gy irradiation (for 5 days); arrows indicate the days of treatment and irradiation (n=8 for each group). (**c**) Quantification of pimonidazole staining in sections of UK-5099-treated FaDu tumor mouse xenografts (n=6 for each group). (**d**) Elapsed time until exhaustion for mice exposed to 3 mg/kg 7ACC2 or vehicle (DMSO) as determined by the refusal of mice to remain on the treadmill belt (n=10 mice per group). Data are represented as mean ± SEM. Significance was determined by student's *t* test. *p<0.05.

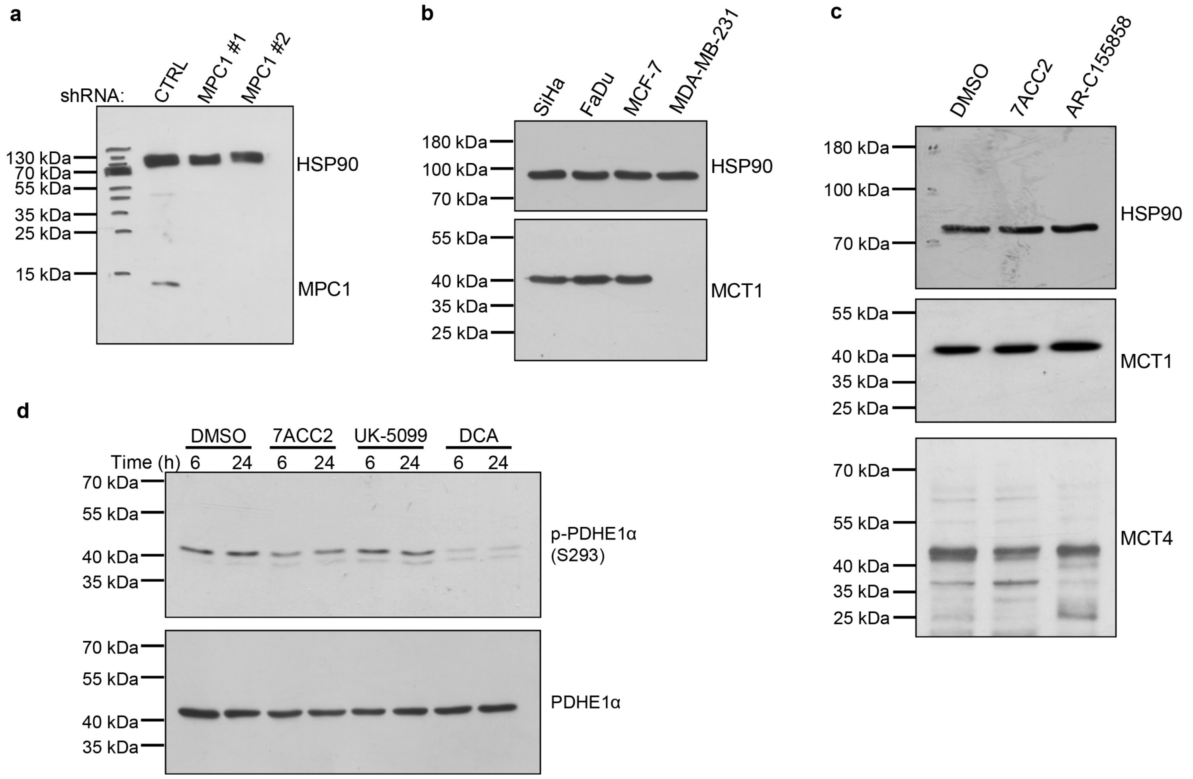

Supplementary Figure 7: Scans of uncropped gels shown in the main text and in the Supplemental information. Uncropped versions of western blots from Figure 5j (**a**), Supplementary Figures 1a (**b**), 1d (**c**) and 4c (**d**).

Supplementary Note 1

Chemical structure of 7ACC2

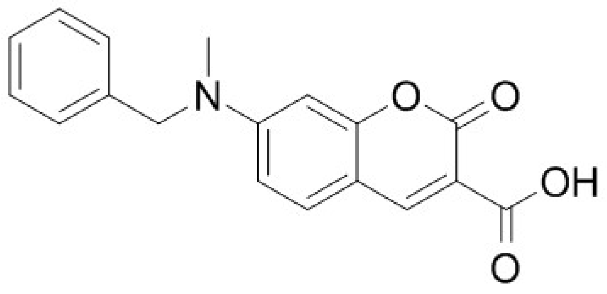

Toxicity and Pharmacokinetics data.

At the dose used in our *in vivo* experiments (3 mg/kg), we did not observe neither significant weight loss nor abnormal lesions on gross necropsy. In our attempts to evaluate different routes of administration, we found that besides intraperitoneal injection (with DMSO as a solvent), i.v. (N-methyl-2-pyrrolidone/hydroxypropyl-β-cyclodextrin) and oral (Tween 80 + Carboxymethylcellulose) administrations were achievable with a similar plasma half-life (4.5, 2.8 and 3.9 hours, respectively). 7ACC2 was further shown to be blood brain barrier-permeant (plasma half-life = 5.0 hours) with a brain/plasma distribution ratio = 1.24. Regardless of the route of administration, at the dose employed in our *in vivo* experiments (3 mg/kg), we did not observe neither significant weight loss nor abnormal lesions on gross necropsy.
