# Supplementary material for: Interruption of lactate uptake by inhibiting mitochondrial pyruvate transport unravels direct antitumor and radiosensitizing effects
Source: Nat Commun. 2018 Mar 23;9:1208. doi: 10.1038/s41467-018-03525-0 (PMC5865202; doi:10.1038/s41467-018-03525-0)
Supplement: Supplementary file 3 — Description of Additional Supplementary Files(PDF 163 kb) [file 41467_2018_3525_MOESM3_ESM.pdf]

Description of Additional Supplementary Files

File Name: Supplementary Data 1
Description: Mass spectrometry data and calculations.
